# Additive manufacturing of patient-specific, biphasic implants with zonal design for regeneration of osteochondral defects–critical evaluation of the work flow from clinical MRI data to implantation

**DOI:** 10.1016/j.mtbio.2025.101858

**Published:** 2025-05-13

**Authors:** Max von Witzleben, Suihong Liu, Philipp Sembdner, Stefan Holtzhausen, Sophia Freya Ulrike Blum, Jörg Lützner, David Kilian, Ute Nimtschke, Michael Gelinsky, Anja Lode, Henriette Bretschneider

**Affiliations:** a Centre for Translational Bone, Joint and Soft Tissue Research, Faculty of Medicine and University Hospital Carl Gustav Carus, Technische Universität Dresden, Dresden, Germany; b Institute of Machine Elements and Machine Design, Faculty of Mechanical Engineering, Technische Universität Dresden, Dresden, Germany; c Institute and Polyclinic for Diagnostic and Interventional Radiology, University Hospital Carl Gustav Carus, Technische Universität Dresden, Dresden, Germany; d University Center of Orthopaedics, Trauma and Plastic Surgery, University Hospital Carl Gustav Carus, Technische Universität Dresden, Dresden, Germany; e Department of Materials Science and Engineering, Stanford University, Stanford, CA, USA; f Institute of Anatomy, Faculty of Medicine and University Hospital Carl Gustav Carus, Technische Universität Dresden, Dresden, Germany

**Keywords:** Additive manufacturing (AM), Magnetic resonance imaging (MRI), Computer-aided design (CAD), Extrusion-based 3D printing, Calcium phosphate bone cement, Hydrogel, Osteochondrosis dissecans

## Abstract

Ideally, the combination of clinical imaging techniques with additive manufacturing processes enables the fabrication of patient-specific regenerative implants that precisely fit into the defect site, promoting native tissue restoration while gradually degrading. Osteochondral defects, affecting both cartilage and subchondral bone in joints are best visualized using magnetic resonance imaging (MRI). In this study, a workflow for computer-aided manufacturing of patient-specific osteochondral implants based on geometrical data obtained from MRI scans was evaluated in a clinically relevant setting. Artificial osteochondral defects were created in femoral condyles of human body donors and scanned with MRI. ‘Computer-Aided Design’ (CAD) models for bone and cartilage components served as basis for designing defect-specific trizonal implants consisting of (i) a bone, (ii) an interlocking, and (iii) a cartilage zone. These implants were fabricated using multi-channel 3D extrusion printing, using a calcium phosphate cement as a bone substitute and an alginate-based hydrogel as a cartilage substitute material - with both materials alternately printed in the interlocking zone. After fabrication, the constructs were implanted into the corresponding defects, and assessed for fit accuracy via clinical imaging. The entire process chain was successfully conducted under near-clinical conditions by an interdisciplinary team of engineers, radiologist and surgeons, during which critical points were identified. Due to the inherent resolution limitations of clinical MRI and extrusion-based 3D printing, inaccuracies in implant fitting occurred; strategies to address these challenges were identified by integrating design tolerances and applying minor intraoperative adjustments.

## Graphical abstract

**Figure:**
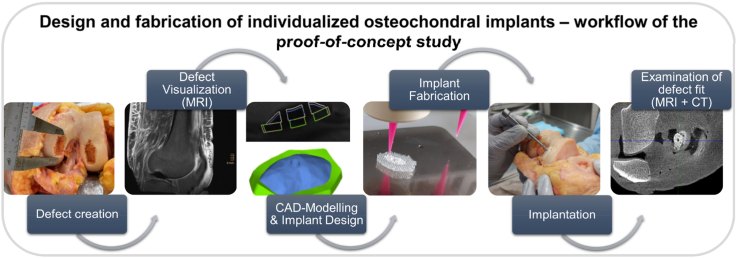

## 1 Introduction

Osteochondral defects, damages to both cartilage and subchondral bone in human joints, are a common orthopedic challenge that often requires surgical intervention at advanced stages. A prevalent example is *osteochondritis dissecans* (OCD), which even affects younger patients or athletes and is characterized by avascular necrosis of the subchondral bone and potential separation of an osteochondral fragment [1]. Treatment approaches vary by stage: conservative management is typically effective in early stages (I and II), whereas surgical intervention is necessary in advanced stages (III and IV) to refix or remove unstable or already detached osteochondral fragments [2]. To achieve healing, the osteochondral fragment should be debrided, and the subchondral sclerosis perforated several times. Cancellous osteoplasty is then usually required to create congruence. This is followed by stable and rotation-proof refixation, preferably using biodegradable implants that are submerged onto the cartilage surface to prevent corresponding cartilage damage [3]. Autologous osteochondral transplantation (OCT) is a treatment option for stage IV defects: Cartilage-bone cylinders are transferred from a less load-bearing region of the joint to the damaged area and fixed in place using the press-fit anchoring principle [4]. For larger defects, autologous chondrocyte transplantation (ACT) is recommended in combination with reconstruction of the bony defect using autologous cancellous bone; ACT involves arthroscopic harvesting of chondrocytes, followed by *in vitro* expansion and implantation either as suspended cells or within a collagen matrix (matrix-associated ACT; MACT) [[5], [6], [7]]. Thus, both approaches have limitations such as donor site morbidity and limited tissue availability. Research efforts are focused on developing alternative strategies for tissue regeneration using engineered osteochondral tissue substitutes [[8], [9], [10], [11]].

The combination of imaging techniques such as computed tomography (CT), magnetic resonance imaging (MRI), and Digital Imaging and Communications in Medicine (DICOM) data with additive manufacturing processes enables the fabrication of patient-specific regenerative implants [[11], [12], [13]]. Ideally, these implants fit precisely into the defect site, promoting native tissue restoration while degrading without compromising the defect site integrity. Extrusion-based 3D (bio)printing is particularly suited for processing a wide range of biomaterials under ambient conditions, including slurries/pastes of mineral phase powders in a carrier liquid and high-viscous (bio)polymer-based hydrogels loaded with cells and/or therapeutic factors [[14], [15], [16], [17], [18]]. This printing technique involves layer-by-layer deposition of material strands according to a predefined pattern, allowing for the creation of volumetric structures with clinically relevant dimensions and open macropores. Multi-channel printing heads facilitate the combination of different biomaterial inks and bioinks (cell-laden hydrogels) within a single construct, effectively replicating the complex hierarchical microarchitecture of tissues and tissue interfaces.

Previously, we introduced multi-channel extrusion printing of a chondrocyte-laden bioink, and a calcium phosphate cement (CPC) paste to produce multi-zonal osteochondral tissue substitutes [19]. The bioink ‘AlgMC’, composed of 3 wt% alginate and 9 wt% methylcellulose, is printable with high shape fidelity and provides a stable, hydrated 3D matrix for embedded chondrocytes after post-printing crosslinking of alginate with calcium ions [20,21]. The oil-based CPC paste, approved for clinical use, transforms from α-tricalcium phosphate to nanocrystalline hydroxyapatite upon contact with water, which resembles the natural bone mineral and thus enables osteoconductivity and osteoclastic resorption [18,[22], [23], [24]]. Using initially simple cylindrical model structures, the osteochondral tissue substitutes were designed with three zones: (i) a cell-laden AlgMC zone for articular cartilage, (ii) an interwoven zone of alternating AlgMC and CPC strands, which provides mechanically stable interlocking between the cartilage and bone region and partially mimics the calcified cartilage, and (iii) a macroporous CPC zone for subchondral bone that should be colonized by cells migrating from the underlying bone and bone marrow [19]. To transfer this three-zonal design to a patient-specific implant for an osteochondral defect, we have recently developed a workflow for computer-aided manufacturing based on geometrical data obtained from MRI scans [25]. MRI offers superior visualization and characterization of osteochondral defects compared to CT, allowing precise identification of defect position, thickness, and orientation of the articular cartilage zone [26,27].

**Fig. 1:**
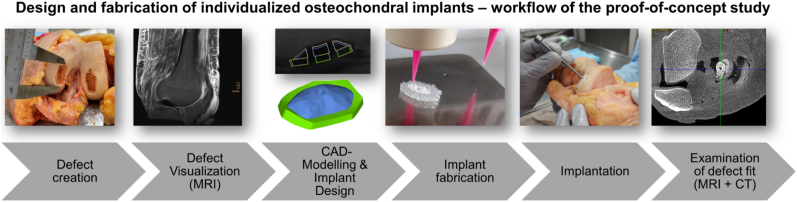
Schematic presentation of the project workflow.

The present study aimed to validate the MRI-guided workflow in a clinically relevant setting by creating artificial osteochondral defects in femoral condyles of human body donors, scanning them with MRI at typical clinical resolution, and developing CAD models for the bone and the cartilage part to design defect-specific implants (Fig. 1). Defect-specific trizonal implants with a bone (CPC), an interlocking (CPC + AlgMC) and a cartilage (AlgMC) zone were fabricated using multi-channel 3D extrusion printing, implanted into the corresponding defects, and assessed for fit accuracy by clinical imaging. Through this multidisciplinary approach, we sought to evaluate the feasibility of the workflow for clinical application and to identify critical considerations within the different steps and their potential limitations.

## 2 Materials and methods

### 2.1 Simulation of clinical cases and practice

*Defect creation*. Osteochondral defects were created manually using a surgical chisel in three knee joints of two human body donors (frozen cadavers). Before defect setting, the bodies were defrosted in the cold room. After defect setting, the knee joints were closed in layers using capsular, subcutaneous and skin sutures, subjected to clinical imaging with MRI and stored at −20 °C until implantation.

*Implantation*. The donor knees with defects were thawed, the suture material was removed, and the knee joints were opened to access the individual defects. For fixation of the inserted implants, a hole was drilled in the underlying bone using the channel(s) in the center of the 3D printed implant. Before SmartNails®, 1.5 mm in diameter and 16–25 mm in length (ConMed Linvatec Biomaterials) were inserted into the drilled channel(s) and placed using the appropriate instrumentation; the heads of the SmartNails® were countersunk up to the CPC part. Afterwards, the knee joints were closed in layers with capsular, subcutaneous, and skin sutures and subjected to MRI and cone-beam CT.

### 2.2 Imaging

For MRI (3 T, Siemens Magnetom Skyra, Siemens Healthineers, Erlangen, Germany), a protocol based on the examination standard for knee MRI (recommended by the AG Musculoskeletal Radiology, German Radiology Society), supplemented by a high-resolution isotropic 3D sequence, was used. All examination parameters are summarized in Table 1. A high-resolution cone-beam CT was acquired of all donor knees (Multitom Rax in Real 3D mode, Siemens Healthineers, Erlangen, Germany) in a standardized manner (0.35 mm slice thickness).

**Table 1:** MRI examination parameters; Abbreviations: PD – proton density, TSE – turbo spin echo, T2 DE3D WE - T2 3D-double-echo steady state (DESS) with water excitation, TE – excitation time, TR – relaxation time, FoV – field of view'.

	PD TSE FS sagittal	PD TSE FS coronal	PD TSE FS axial	T1 TSE sagittal	T2 DE3D WE sagittal
Slice thickness/gap between slices (mm)	3/3.3	3/3.3	3/3.3	3/3.3	0.6/0
TE (ms)	26	27	40	8.9	5.04
TR (ms)	4300	3400	4980	750	14.84
FoV (mm)	150∗150	150∗150	150∗150	150∗150	150∗160
Matrix size	384 x 384	384 x 384	384 x 384	384 x 384	240 x 256
Acquisition time (min)	4:00	3:10	4:24	3:27	5:02

### 2.3 CAD modelling

Extraction of bone and cartilage CAD models from MRI data was performed using segmentation and reference geometry derivation with 3D Slicer V.5.2.2 software (open-source) and the CTinA (CT in Application) software solution, the latter developed at the Chair of Virtual Product Development at TU Dresden [25,28]. Solid models were then generated using SolidWorks (Dassault Systèmes, France).

Prior to printing, the corresponding 3mf files were sliced into a G-code using the corresponding control software of the utilized BioScaffolder 3.1 (GeSiM mbH, Radeberg, Germany).

### 2.4 Preparations for printing

The AlgMC ink was prepared as described previously [20,21]. In brief, 3 wt% alginic acid sodium salt from brown algae (Sigma-Aldrich, Germany) and 9 wt% methylcellulose powder (4000 cP, Sigma Aldrich, USA) were autoclaved (121 °C for 20 min in a Systec D-23 table-top autoclave). Subsequently, both polymers were dissolved in phosphate buffered saline (PBS; Thermo Fisher, USA) and left at 4 °C overnight. To produce the sacrificial MC ink, 10 wt% methylcellulose was autoclaved, dissolved in PBS and stored at 4 °C overnight. Prior to 3D printing, the AlgMC and MC inks were centrifuged with 1500 rpm for 5 min to remove trapped air. The CPC paste (Plotter-Paste-CPC), a printable calcium phosphate cement formulation based on α-TCP [18,22], was provided by INNOTERE GmbH (Radebeul, Germany).

Based on the adjusted bone CAD models, external support structures were pre-fabricated with a stereolithographic printer (SLA, Mars 2 Mono, ELEGOO, China) using a generic photopolymer resin (Tough Resin 2.0, ANYCUBIC, China).

### 2.5 3D printing and postprocessing of implants

The multi-channel extrusion printer BioScaffolder 3.1 was equipped with three cartridges and conical nozzle tips with an inner diameter of 250 μm or 410 μm (Nordson EFD, Germany) were used for fabrication of the defect-specific implants. Glass slides served as printing bed. Corresponding 3mf files were uploaded into the GeSiM software and models were printed with a 90° layer-to-layer orientation, either including the sacrificial MC support (with an infill offset of 0.1 mm for the MC support) or into the external support structures. The printing parameters are summarized in Table 2.

**Table 2:** Printing parameters applied for implant fabrication in each experiment.

Printing parameters	K1 + K2	K3
Inner nozzle diameter (μm)	410	250
Applied pressure (kPa)	CPC: 85-90	CPC: 110-135
	AlgMC: 120-125	AlgMC: 80-100
	MC: 180-290	MC: 220-240
Printing speed (mm/s)	5.5	CPC: 4-5
		AlgMC: 5.5
		MC: 5
z-offset (mm)	0.3	0.2
Strand height (mm)	0.3	0.2
Strand distance (mm)	0.9	0.9

After printing, trizonal implants printed onto the pre-fabricated support structures were moved to a water-saturated atmosphere at 37 °C to facilitate the self-setting of CPC. After 60 min, alginate crosslinking was induced by addition of approx. 0.5 mL 100 mM CaCl_2_ onto the upper part of the implants consisting of AlgMC; after additional 10 min, approx. 1 mL CaCl_2_ solution was applied to ensure fully crosslinking. Implants were kept in 100 % humidity overnight to ensure full setting of the CPC, and then the external support structure was carefully removed. Afterwards, the implants were transferred into 50 mL-tubes containing 40 mL of 25 mM CaCl_2_ in Hanks’ balanced salt solution (HBSS, Sigma-Aldrich, Germany) for CPC hardening and stored for 3 days at 37 °C until implantation. Trizonal implants with printed sacrificial MC support were moved to water-saturated atmosphere at 37 °C and incubated for 30 min before 100 mM CaCl_2_ was added for alginate crosslinking. After 30 min, the implants were transferred into 50 mL-tubes containing 40 mL of 25 mM CaCl_2_ in HBSS to dissolve the MC support while stabilizing the Alg and harden the CPC for 3 days storage at 37 °C until implantation.

## 3 Results

### 3.1 Defect creation and clinical imaging of the defect region

**Fig. 2:**
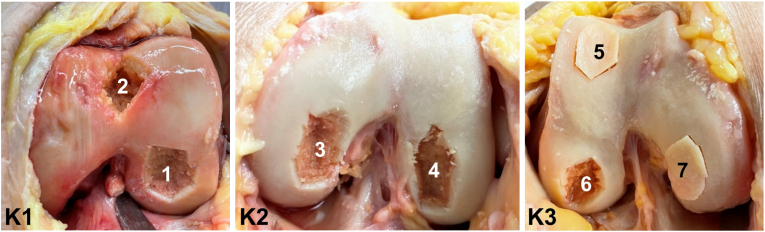
Defects (1–7) created in the three knee joints (K1-3); defects no. 5 and 7 with osteochondral fragments kept in place.

Seven osteochondral defects of different sizes (1.00–1.74 cm^2^, average defect size: 1.34 cm^2^) were created in three knee joints (K1, K2, K3) from human body donors (Fig. 2). The defects were set in the medial or lateral femoral condyle at different distances from the intercondylar femoral fossa or at the trochlear femoris. Advanced osteochondral defects stage III according to Bruns [2] with interruption of the cartilage surface and osteochondral fragment *in situ* (2/7: defect no. 5 and 7) as well as stage IV with detached, free osteochondral fragment and empty defect area (5/7: defect no. 1–4, and 6) were generated (Table 3).

**Table 3:** Size and location of the seven defects created in the three knee joints.

Knee	K1		K2		K3		
Defect No.	1	2	3	4	5	6	7
Size (cm^2^)	1.62	1.0	1.17	1.74	1.08	1.41	1.39
Maximal defect length & width (mm)	19 × 13	14 × 9	10 × 16	9 × 19	20 × 14	11 × 18	12 × 19
Location	lateral femoral condyle	trochlea	medial femoral condyle	lateral femoral condyle	lateral femoral condyle	lateral femoral condyle	medial femoral condyle
Stage according to Bruns	IV	IV	IV	IV	III	IV	III

**Fig. 3:**
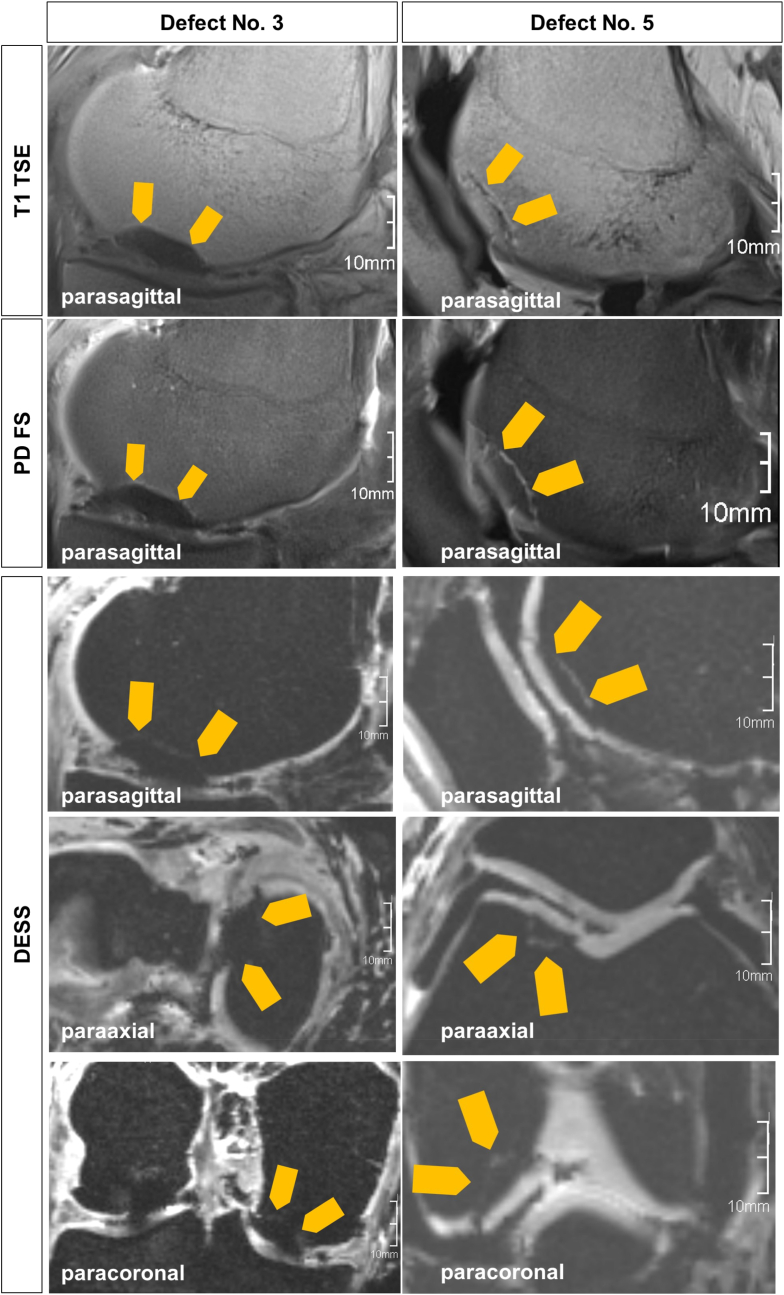
Exemplary imaging of the empty defect No. 3 (K2) and defect No. 5 (K3) with remaining osteochondral fragment in the MRI. T1 turbo spin echo (TSE) sequence, proton density fat saturation (PD FS), double-echo steady-state (DESS). The orange arrows mark the defect. (For interpretation of the references to colour in this figure legend, the reader is referred to the Web version of this article.)

The osteochondral defects could be well visualized in comparable quality to the current clinical situation, as exemplified by defects No. 3 and No. 7 (Fig. 3). The additional high-resolution isotropic 3D sequence with the possibility of 3D reconstruction was helpful for the precise determination of the defect geometry and cartilage-bone interface.

### 3.2 Segmentation of the defects

Through segmentation of the MRI data of the defects, corresponding CAD models were successfully generated. The models need to mimic the original curvature of the joint to enable a smooth movement of knee joint post-surgery. To realize this with the layered principle of 3D printing while allowing a multi-material print, each defect was split into two parts: a bone and a cartilage part.

Therefore, image data from *PD TSE FS* series in *sagittal, coronal*, and *axial* orientations (depending on the defect position) were utilized for segmentation. Within the CTinA software, relevant defect areas were identified, with at least three images selected for boundary edge segmentation for the model design. The distance between each image was 3.3 mm, and to create a closed 3D model, additional virtual images were required, generated by interpolating between the existing images (see Supplementary Video 1 and Supplementary Video 2 for K3). Within each image, three boundary curves were manually outlined to segment only the pertinent bone areas for model creation and reference points were manually set along the visible defect edges at the bone-cartilage and cartilage-air interfaces. The number of these points varied based on the complexity of the shape, with more points set for complex shapes to ensure accurate boundary curves representing the contour and gradient transition as effectively as possible. All boundary curves were defined with an extension of approximately 5–10 mm beyond the defect area to facilitate clean intersection of individual boundaries during model creation. In cases where cartilage contour details were insufficient, contours were extrapolated tangentially to achieve an aesthetically and functionally adapted model for accurate reproduction of knee joint rolling geometry (J-curves), (Fig. 5A). Finally, the *T1 TSE sagittal* and *T2 DE3D WE sagittal* series were employed to validate the agreement of the defect boundaries with the 3D-model.

Subsequently, each of these curves were imported into SolidWorks (Fig. 5B), where loft surface functions were used to define specific guide curves for creating free-form surfaces in three areas (Fig. 5C): 1) outer cartilage contour, 2) bone-cartilage interface, and 3) inner bone contour. These surfaces were then intersected and combined to form two solids representing the bone and cartilage model after the drilling holes were inserted (Fig. 5D–F).

### 3.3 Design of the implants

Before printing and implantation, these models were then adjusted from a clinical perspective to optimize their design for good clinical practice.

1. The outer contour of the cartilage part was enlarged by 1–2.5 mm compared to the cartilage defect to ensure a close implant fit, ideally resulting in a closure of the cartilage surface without any holes. Due to the softness of the hydrogel, the cartilage part can be easily adjusted intraoperatively in case of over-enlargement.
2. Due to the resolution of the MRI of 0.39 mm and the gap between slices of 3.3 mm, small protrusions within the defect (<500 μm) cannot be seen in the pre-operative MRI data. On the other hand, the bone part of the implant may be smaller than the bone defect as small gaps (≤1 mm) can be bridged by the host bone; adjustments of the bone defect area are common practice for treating osteochondral defects. Consequently, additional bone models with a 0.5 mm negative offset in the x, y, and (optionally) z directions were designed.
3. The drill channel(s) required for the SmartNail® fixation should be as orthogonal as possible to the bone surface. Depending on the size of the model, one or two channels were designed to ensure stable fixation of the implant.
4. To prevent reduction in cartilage thickness or potential ossification, the bone part should not protrude into the cartilage part; hence, the interlocking layers for the trizonal design must be located within the bone model.

**Fig. 4:**
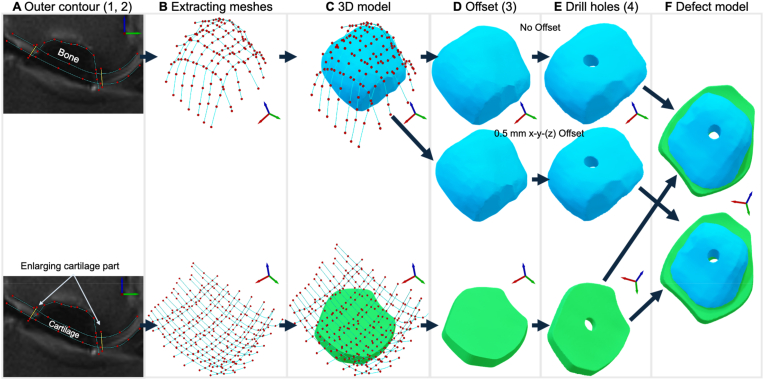
Modeling process using CTinA exemplarily shown for implant No. 6. (A) Definition of the contours (blue curves) and boundaries (yellow line) in the MRI sectional image, (B) extraction of the contour meshes depending on the reference points, (C) Contour package for deriving the bone and the cartilage model, (D) realizing the offset, (E) setting the holes for the SmartNail® fixation, and (F) combining parts. (For interpretation of the references to colour in this figure legend, the reader is referred to the Web version of this article.)

These adjustments were added to the workflow of 3.2. (Fig. 4). In brief, the defect models were extracted by setting referencing points within the MRI data resulting in a mesh, which then was extracted and used to model the corresponding bone and cartilage part (Fig. 4A–C). The enlargement of the outer cartilage boundary is depicted as yellow lines in Fig. 4A. By utilizing the outer contours and surface functions, the respective 3D models were developed, the offset and the holes were included (Fig. 4C, D, E) before the respective parts were combined in one 3mf file (Fig. 4F).

**Fig. 5:**
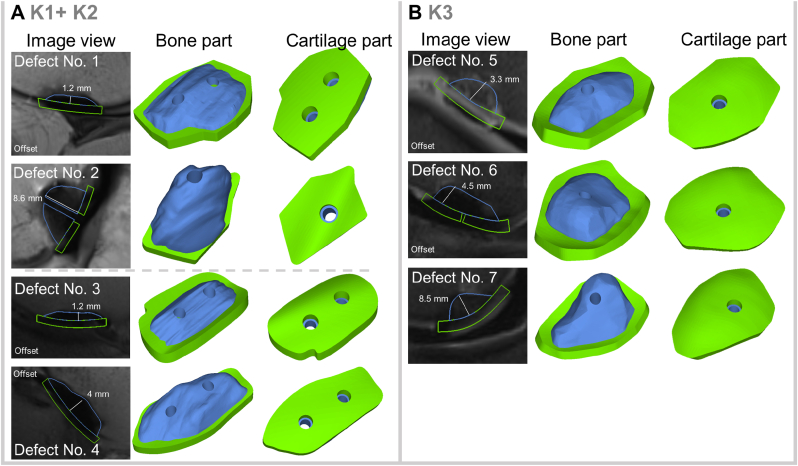
Overview of the designed models of the actual implants for K1, K2 (A) and K3 (B).

These steps were then performed for each defect. The number of drill holes was defined by the x-y-area of the bone defects for K1 + K2: when the area was larger than 1 cm^2^ two holes were designed. However, the implantation of the scaffolds into K1 and K2 showed that these holes increased the fragility of the CPC part and that one SmartNail® is sufficient even for areas larger than 1 cm^2^. Therefore, only one hole was designed within the models for K3. A similar effect was observed for the offset, so instead of a bone offset in x-y-z direction, a bone-offset in x-y direction was set to ensure a stable CPC structure. An overview of each implanted scaffold model is depicted in Fig. 5.

### 3.4 Fabrication of implants realizing a trizonal design

**Fig. 6:**
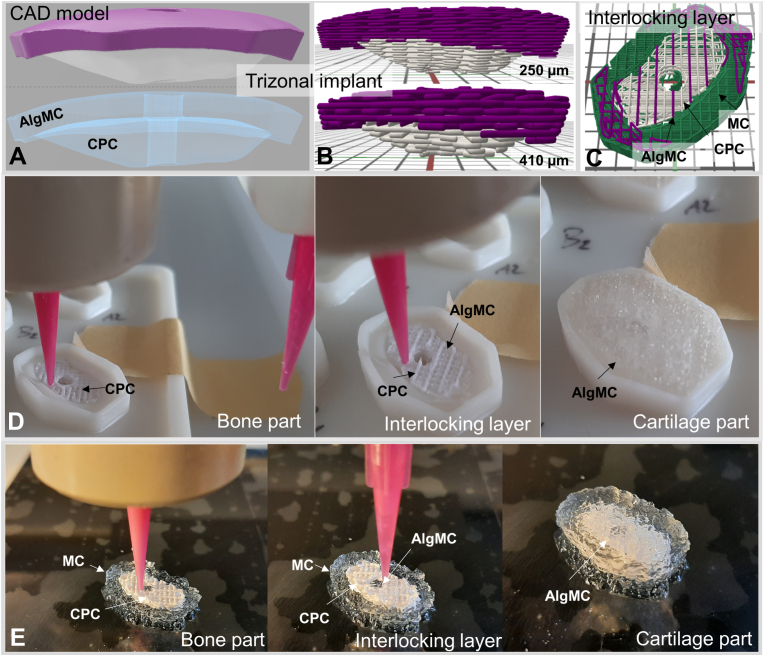
Display of the scaffold fabrication process exemplarily for implant No. 5. CAD-models of the defects (A, transparent version to highlight the designed channel) with a cartilage part (purple) and a bone part (white) were sliced into different number of layers depending on the nozzle outlet diameter (B, 410 vs 250 μm). Within this step, the interlocking layers were designed with an alternating printing pattern between AlgMC and CPC (C), whereas the green strands represent the MC support. The printing process was executed with a multi-material printer by either printing into prefabricated support structures (D) or by simultaneously printing sacrificial hydrogel support structures based on 10 % MC (E). Both image series first show the printing of the CPC part (left), followed by the printing of the interlocking layers (middle), and finally, the completed print outcome (right). (For interpretation of the references to colour in this figure legend, the reader is referred to the Web version of this article.)

Based on the previously established printing principle [25], the corresponding 3mf files were sliced with a defined strand pattern, number of layers and optionally with a MC support structure (Fig. 6A–C). The printing process was carried out in a single step using a multi-channel print head. To prevent delamination of the cartilage and the bone part upon surgery and to avert CPC strands from protruding into the contemplated cartilage part, four interlocking layers were designed by defining an alternating positioning of CPC and AlgMC strands within the bone part (Fig. 6C).

The convex bone part was either supported by a pre-fabricated support structure [29,30] or by a sacrificial hydrogel which consisted of MC and was printed simultaneously with the respective implant [25,31]. For each defect, implants with and without −0.5 mm offset were printed. Thus, defect-specific trizonal implants were successfully fabricated by 3D printing the CPC-based bone part first, followed by printing the interlocking zone of CPC and AlgMC and finally the AlgMC cartilage part (Fig. 6D–E). Printing the CPC part first facilitated the removal of the implants from both, the prefabricated external support structure and the sacrificial MC support, without the risk of damaging the soft AlgMC hydrogel of the cartilage part.

SLA printed support structures were pre-fabricated in two separate halves, based on the CAD models cut in half in the x/y plane, to facilitate the removal of the printed implants from the support structure during post-processing (Supplementary Fig. S1). They were placed on top of the printing bed (Fig. 6D) and the corresponding scaffold centers were configured within the GeSiM software. Of course, each removal or exchange of these structures required a new configuration step. In contrast, the MC supported scaffolds were directly printed in the same process onto a glass printing bed (Fig. 6E).

For the implants 5–7 (K3), the nozzle diameter was decreased from 410 μm, used for the defects 1–4 (K1, K2) according to our previous work [25], to 250 μm to realize smaller details at the scaffold outline and to achieve a high detailed replication of the CAD model (Fig. 6B). However, this step doubled the total number of layers, ranging from 30 to 66, depending on the defect depth. This allowed for the printing of a higher number of pure CPC layers before printing the interlocking layers, which increased the overall stability of the printed constructs, relevant especially for thin defects with a flat bone compartment. However, the increase in the number of layers also caused an increase in printing times, ranging from 7 (Defect No. 5) to 53 min (Defect No. 7) depending on the defect depth. Additionally, printing the sacrificial MC support structure approximately doubled the printing time compared to printing procedures using the SLA printed external support structures.

On the other hand, the moisture in the MC hydrogel support initiated the setting process of the CPC already during the printing process, accelerating the overall setting and increasing initial stability of the implants after the printing process. In contrast, the SLA printed, external support structures were printed in two halves to enhance humidity accessibility and ensure thorough setting of the CPC. Nevertheless, the incubation time had to be prolonged from 30 min, which was applied for CPC with MC support, to overnight to achieve sufficient hardening for mechanical removal from the support structure.

### 3.5 Implantation and clinical imaging

**Fig. 7:**
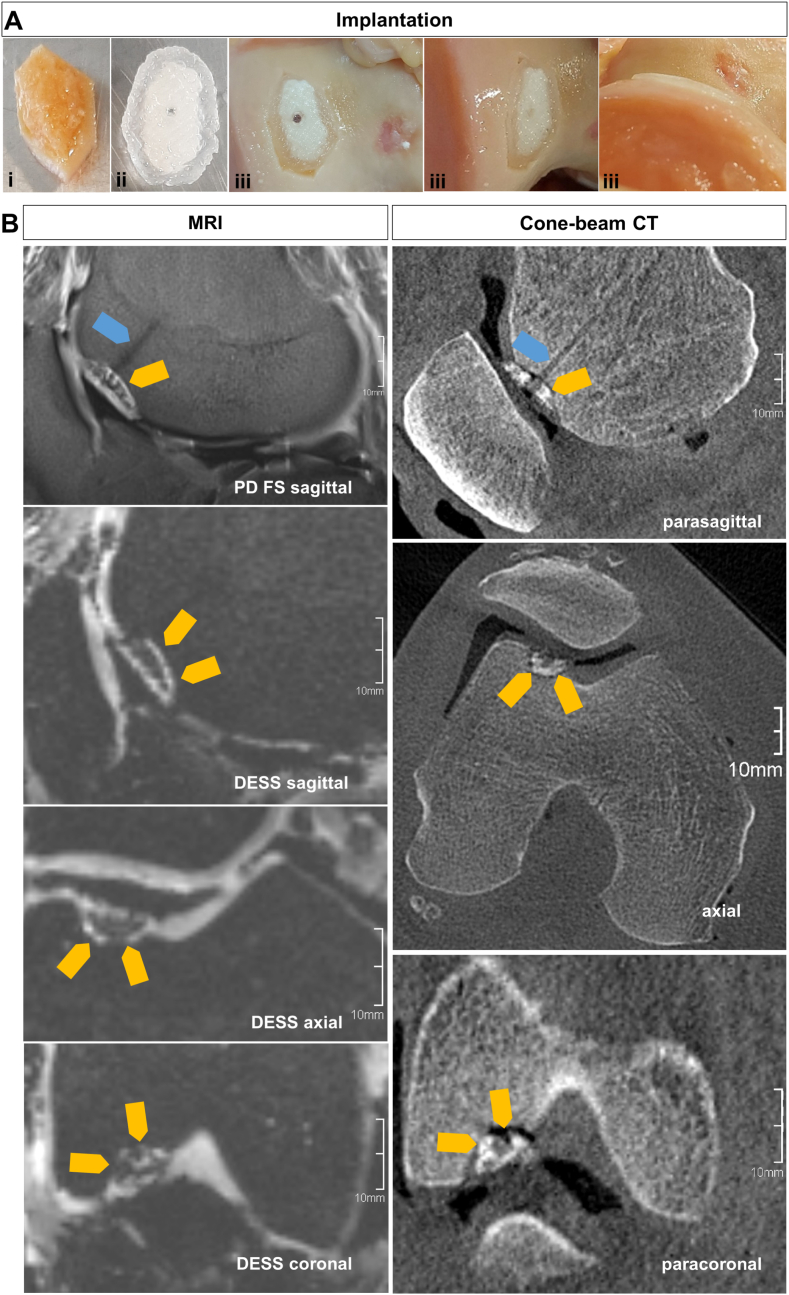
Implantation **(A)** and post-implantation imaging **(B)** exemplarily shown for defect No. 5. **A:** Osteochondral fragment (i) removed prior to implantation, the implant structure before (ii) and after (iii) implantation. Clinical images showing the inserted implant fixed with a SmartNail® through a drilled central hole. **B:** Three-dimensional reformations along the implant in high-resolution MRI und CT. The orange arrows mark the defect filled with the implant, the blue arrows the channel with the SmartNail®. (For interpretation of the references to colour in this figure legend, the reader is referred to the Web version of this article.)

During implantation of the trizonal implants, specimens of the different variants (the pool of implants is summarized in Table 4: w/and w/o offset, fabrication with prefabricated external support vs. fabrication with sacrificial MC support) were first placed into the defect and their fitting was visually evaluated. The implant with the best fit, i.e., sitting in the defect without tipping, completely filling the defect, and with the hydrogel part flush with the adjacent cartilage, was then fixed using SmartNail(s)®. In Fig. 7A, the implantation process is exemplarily shown for defect No. 5. The used implant was printed with an −0.5 mm x-y-offset of the bone part and was fabricated with the MC hydrogel support. Over-enlargement of the cartilage part consisting of AlgMC was cut with scalpel. In 4 out of 7 cases, an over-extruded CPC strand end (fabrication artefact), preventing a satisfactory fit into the defect, was cut off manually which was easily possible.

**Table 4:** Overview of the pool of implants and the final implanted constructs based on a visual best fit of the involved clinicians. Following abbreviations are used to list the different implants: “±0” for no offset, “-0.5” for offset (in mm) in the bone part, SLA for the usage of external SLA-printed support structures and MC for the sacrificial hydrogel support.

Knee	K1		K2		K3		
Defect No.	1	2	3	4	5	6	7
Pool of Implants	SLA ±0/SLA -0.5		SLA ±0/SLA -0.5		SLA ±0/SLA -0.5		
			MC ±0/MC -0.5		MC ±0/MC -0.5		
Offset directions	x-y-z				x-y		
Nozzle Ø (μm)	410				250		
Number of CPC-layers	3	21	3	10	13	18	34
Implanted	SLA -0.5	SLA ±0	SLA -0.5	SLA -0.5	MC -0.5	SLA -0.5	MC ±0

Five out of 7 utilized implants were manufactured with the support structure and 2 out of 7 using the sacrifical ink. In addition, an offset was advantageous for 5 out of 7 implants; only 2 out of 7 implants without offset were preferred in terms of accuracy of fit (Table 4). The implants No. 1, 2, and 6 were broken during implantation, most probably because of an insufficient fit of the osseous part. This resulted in tilting and oblique insertion of the implant with a lack of implant-bone contact in some areas. The fracture occurred when the SmartNail® was inserted.

After implantation, the contact with the subchondral bone and the fit to the cartilage and the joint surface were assessed by employing CT and respectively MRI scans (Fig. 7B). In CT, CPC can be identified as hyperdense to cortical bone (see Supplementary Video 3 for K3). In some cases, the hydrogel layer could be delineated as well when it was directly covered by air in the joint space. In MRI, CPC is hypointense to the bone in all sequences and the hydrogel showed a high signal intensity, which was comparable to that of the cartilage. The air in the joint space of the donor knees minimally impaired the delineation of the implant when localized directly adjacent to the hydrogel or CPC.

### 3.6 Assessment of fitting

**Fig. 8:**
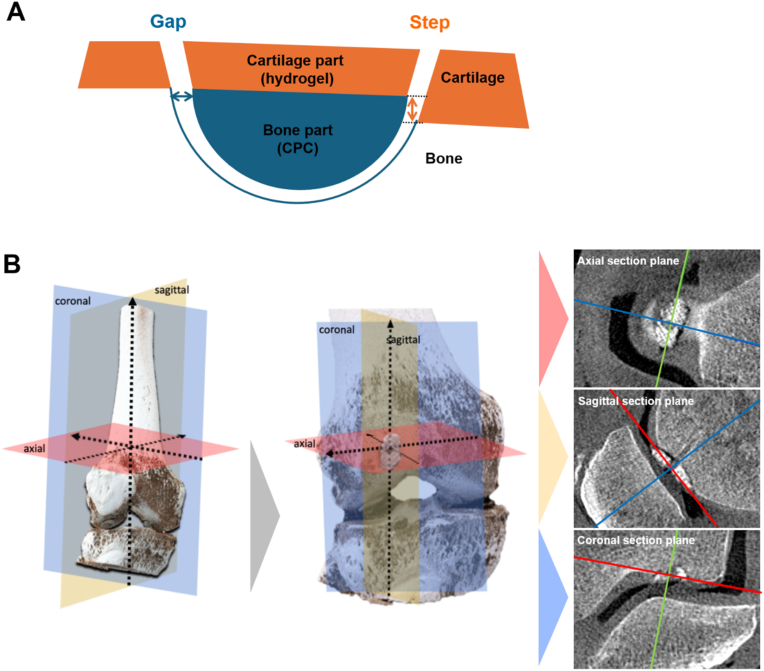
Assessment of fitting of the implants. (A) The schematic shows the difference between the measured gap (blue arrow) and step (orange arrow). The bone part comprises both the pure CPC layers and the interlocking layers of alternately printed CPC and AlgMC at the interface to the cartilage part. (B) Determination of the axes for measuring the fit between CPC and bone at 4 measurement points. The axes were placed orthogonally to each other and aligned along the long axis of the implant: depiction in axial, sagittal and coronal sectional plane. (For interpretation of the references to colour in this figure legend, the reader is referred to the Web version of this article.)

From a clinical perspective, both the contact with the subchondral bone and the smooth transition between the cortical bone and the articular cartilage, which should ideally correspond to the thickness of the surrounding cartilage tissue, are crucial. Using the CT images, fitting of the bone part of the implant was determined as illustrated in Fig. 8A, by measuring (i) the gap size between the implant and the subchondral bone (first row of Table 5) and (ii) the step of the bone part of the implant to the adjacent bone (second row of Table 5). With the step value, it was assessed whether the upper surface of the CPC part followed the contour of the cortical bone interface without protruding into the cartilage part. Ideally, this value is 0; negative values indicate a lower level of the implant, and positive values indicate a protrusion of the bony part of the implant.

**Table 5:** Measuring the implant fit using CT and MRI.

Knee	K1		K2		K3		
Defect No.	1	2	3	4	5	6	7
CPC: gap in defect (mm)							
Anterior	Dislocation	1.3	0.8	0	1	0.6	0.9
Posterior	0.9	0.6	0	1	0	0	0
Medial	0	0.8	1.8	0.7	0	0	0
Lateral	1.3	1.7	0	0	0.6	0.7	1.4
**CPC: step to the cortical bone (mm)**							
Anterior	Dislocation	0.4	1.5	1	1.4	0.5	1.7
Posterior	0	0	1.6	2	0	1.4	0
Medial	2.4	−2.4	1.8	1.3	1.2	2.3	0.8
Lateral	0	−1	1.3	0.9	−0.7	2.2	1
**Hydrogel (analogous to MOCART-Score)^#^**							
^#^Volume and integration; max. 35 points	30	10	30	30	35	25	30

Both the gap and the step values were determined at the anterior, posterior, medial, and lateral margins. For alignment, two orthogonal axes were placed within the CT images tangential to the surface of the CPC (Fig. 8B). The main axis was aligned collinearly with the long axis of the implant, while the second axis was placed orthogonally at the center of the main axis, determining the other measurement points. In this manner, each of implants’ osseous fit was quantified with eight measurement values – gap and step value at each of the four measurement points.

The gaps between the subchondral bone and the implant deviated between 0 and 1.8 mm for K1 and K2, with an average value of 0.73 mm. For K3 the gap size decreased to 0–1. 4 mm, 0.43 mm on average, without gaps in posterior and medial for all three implanted constructs. Best fitting implants with respect to the measured gap were No. 4, 5, and 6 with ≤1 mm, followed by implant No. 7 and 3 with a gap of >1 mm only laterally or medially (Table 5).

The step to the adjacent bone deviated from −2.4 to 2.4 mm for K1 and K2 and from −0.7 to 2.3 mm for K3, with a positive step for most implants what means a protrusion of the CPC part of the implant into the adjacent cartilage. Implants No. 3 and 6 showed the highest and implants No. 5 and 7 the lowest average deviation from the step value of 0 (implant No. 1 was excluded due to partial dislocation). Interestingly, implants No. 2 and 7, which were manufactured without offset, showed neither the lowest gap values nor the highest step values (Table 5).

The fit of the AlgMC hydrogel and the cartilage was quantified in the MRI images analogous to the MOCART score, whereby the volume of the cartilage defect filling compared to the native cartilage and the integration into adjacent cartilage were evaluated [32]. The MOCART score is used for the assessment of cartilage repair tissue after different cartilage repair techniques in MRI. Five out of 7 implants were able to achieve over 85 % of the possible score (more than 30 as listed in Table 5).

## 4 Discussion

This study aimed to validate the MRI-guided workflow for additive manufacturing of patient-specific implants with zonal design for regeneration of osteochondral defects, as proposed in our previous work [25], under conditions close to the clinic. First, we defined the requirements for the approach from the clinical perspective.

1. For MRI, the clinically used settings must be applied for a realistic scenario; the use of a high-end research MRI with a very high resolution is not available in clinical application.
2. Implantation and fixation of the implant in the defect must be simple, efficient and reliable, and clinically established solutions should be preferred.
3. The CPC part of the implant with its porous structure is intended to allow ingrowth of new bone tissue, as shown in previous studies [33,34], from the subchondral bone and remodeling over time. Ideally, it should be in contact with the subchondral bone, however, a gap of ≤1 mm is considered acceptable based on (pre)clinical experience [35,36].
4. At the bone-cartilage interface, the zonal implant must fit as precisely as possible; any protrusion of the bone part into the cartilage area must be avoided.
5. The hydrogel part of the implant must completely fill the volume of the cartilage in the defect and be integrated into the adjacent cartilage without gaps. It must not protrude from the cartilage surface and must have a homogeneous structure without irregularities.

Based on these requirements, we carried out an initial test on a pig femur from the slaughterhouse. Usually, 2D-sequences are primarily used for the radiological evaluation of osteochondral defects. The test revealed that the slice gaps of MRI imaging led to considerable inaccuracies in the design when only using on plane. Therefore, we opted for an additional MRI sequence (T2 DE3D WE) and designed the implants with the help of all three planes. Additionally, we used an offset in x-y-z direction of −0.5 mm in the CPC part of the implants, which should reduce the impact of such inaccuracies and is within the tolerance of 1 mm, which was defined as an acceptable gap distance. Furthermore, this initial trial indicated that the implant fabrication using methylcellulose as a sacrificial support according to our previous work [25] may also carry an inherent risk of inaccuracy due to gravity acting on the not yet stabilized materials during fabrication, which can lead to minimal deformation of the implant. Therefore, we decided to additionally use external structures pre-fabricated by SLA [29,30] to support the convex bone part, to ensure shape maintenance during printing and before the CPC hardens (Fig. 6). The use of a pre-fabricated SLA support structure increases the shape accuracy of the CPC surface in contact to the support up to the accuracy of the manufacturing process of the support structure [29,30]; according to the manufacturer, the accuracy of the SLA printer is 50 μm, however we faced inaccuracies of a few hundred micrometers due to the post-processing of the materials. Hence, these inaccuracies are highly dependent on the printer, material, print settings and post-processing protocol used. The workflow was optimized to the best of the authors' knowledge.

The press-fit technique proposed in our previous work [25] for implantation using an individual fixation adapter to the surgical plunger shaft, which was designed taking into account the outer shape of the implant surface (cartilage site) and manufactured using SLA, did not prove to be a favorable method: The soft hydrogel was damaged during the harsh procedure and the brittle CPC had a high risk that parts of it break during press fit insertion into the host bone. As an alternative, the SmartNail®-fixation method, which is well established in the clinic, was successfully tested. However, intraoperative drilling through the implant again carries the risk of breaking the CPC. We have therefore decided to design the implants with channels that serve as a guide structure and thus enable drilling into the host bone during the operation as well as SmartNail® insertion.

The design of the implants (see section 3.3) based on the CAD models obtained from the MRI data segmentation considered the above-mentioned requirements from the clinical perspective as well as the observations from the preliminary test on the pig femur. Moreover, further adaptations have been made during the study: The implantations into K1 and K2 were performed within one week, those in K3 later point. Therefore, the experience of the simulated operations in K1 and K2 were used to optimize the implant fabrication for the K3 defects to enhance the implantation accuracy. Furthermore, the applied force on the constructs when using SmartNails® resulted in the breaking of the thin scaffolds No.1 and No.3 (in the first trial). Both were designed with two SmartNails® to ensure rotationally stable anchorage with one SmartNail® per cm^2^ and a channel diameter of 2 mm to ensure the insertion of the SmartNails® without damaging the CPC part. As countermeasure, the implants for K3 (No. 5–7) were designed with only one channel, even if the size of the implant clearly exceeded 1 cm^2^ in surface area. Furthermore, the CPC part was strengthened to prevent fracture by usage of a smaller printing nozzle diameter (250 μm), that resulted in an overall higher number of layers and with keeping the number of interlocking at four, the number of pure CPC layers increased (Table 5). To close strengthen the CPC part further, the offset in z-direction was deleted. Advantageously, the usage of a smaller nozzle diameter should allow for a more precise outline, which might have resulted in smaller gaps between scaffold and tissue.

Overall, we identified the following critical points of the approach:

*Interface clinical imaging/modeling.* Maybe the most critical point is the slice gap of clinical 2D MRI data which can result in inaccuracies of the models (compare to Supplementary Video 1 and Supplementary Video 2 for K3). With the advent of 3D MRI sequences, like the high-resolution isotropic DESS sequence [37] without slice gap that we used additionally, this obstacle might be overcome. However, the contrast between cartilage and bone was lower compared to the 2D sequences (Fig. 3). In the third experiment (K3 implants), the boundary edge segmentation for the model design (by manual setting of reference points along the visible edges of the bone-cartilage and the cartilage-air interface) was performed in an interdisciplinary team of engineer, radiologist and surgeon; interdisciplinary teamwork is an aspect of optimization of the fitting that should not be underestimated. Especially helpful was the segmentation of the osteochondral defect in all three planes allowing for more accuracy despite the existing slice gaps in the MRI images. A big advantage of the clinically used 2D sequences is the comparatively high contrast between cartilage and bone which facilitates a more accurate planning of CPC and the AlgMC phase. The remaining uncertainty in the models, which cannot be avoided, should be counteracted by integrating acceptable tolerances. Accordingly, we have introduced the offset of −0.5 mm in the stiff CPC part and enlarged the flexible cartilage part which can be easily trimmed during implantation.

*Implant fabrication.* The decrease of the nozzle diameter supported a better fitting outline for both investigated support structures but prolonged the printing time significantly. Hence, the employed materials should be stable for up to 1 h, a fact that aggravates bioprinting dramatically, as the bioink resting time should not exceed 45 min to prevent a major cell death from drying out [38]. Using an external SLA-printed support in contrast to print the support structure in a single process helped to reduce the printing time, but a precise positioning of the external structures on the printing bed was detrimental for a precise implementation of the CAD model. On the other side, printing the MC support structures simplified the procedure as the complete printing process happened on one printer. Besides that, a key challenge of the MC printing process was to adjust and maintain the swelling of the hydrogel strands to the dimensions of the non-swelling CPC strands to ensure an identical z-height within each layer.

Comparing the implantation fit of both support structures suggests the usage of SLA printed structures for thin implants whereas the MC support seems to be favorable for deeper defects. The reasons for that could be a slight dispositioning of the prefabricated support structures, so that the deviations increased with increasing number of layers, whereas the water in the MC support pre-hardened the CPC strands and with that especially strengthened the lower CPC layers of large defect sizes, which tend to collapse inside the SLA support structures.

For clinical translation, the overall timeframe is critical. Imaging and CAD processing can be completed within 24 h, depending on staff availability. However, the setting of the CPC component extends acellular fabrication to three days. If autologous cells are used, harvesting and expansion will significantly prolong the process, but the time required would be similar to standard ACI/MACI protocols. As bioprinting can be performed within hours of cell harvest, implantation can follow immediately after CPC setting, the overall fabrication time would be similar to the clinical gold standard. However, the post-processing step may become more complex when full cell functionality must be preserved.

Previous studies have demonstrated feasibility and shown that elevated Ca^2+^ concentrations and alkaline pH shifts exert only a transient, localized effect on cells at the CPC–bioink interface [19,25,39]. An initial decrease in cell viability is observed at this interface, but cell migration during subsequent cultivation compensates for this effect [39]. Thus, using the fabrication regimen established in earlier work, it is possible to produce implants with chondrocytes embedded in the hydrogel component.

*Implantation.* A critical point was to find the correct position of the implant inserted into the defect. The fit was difficult to assess intraoperatively, as the bone part of the implant, which is important here, is covered by the cartilage part and cannot be evaluated in depth (Fig. 8A). Control by means of an intraoperative CT scan would prolong the operation time and mean additional radiation exposure for the patient and is therefore questionable. The use of a template with contact to the femoral condyle could be a solution to mark the exact alignment of the implants in the x-y direction. Small corrections were made to some implants to improve the fit intraoperatively – small CPC protrusions or strand ends were trimmed to compensate for inaccuracies in the design or imperfections during the fabrication process – such a flexibility is inevitable. In a few cases, the soft hydrogel was laterally trimmed to optimize the fit to the cartilage part. We never observed any undersizing of the cartilage part. In the unlikely event that the cartilage zone should not fully cover the defect, additional AlgMC hydrogel could be injected to fill any remaining gaps.

Thus, we have achieved an acceptable fit regarding a gap between CPC and subchondral bone of ≤1 mm for implants No. 4, 5, and 6 and to a limited extent for implants No. 3 and 7 (one side >1 mm). A critical point is the step formation between the CPC and the cortical bone, which should be < 1 mm for active patients [40] but is < 2 mm for the non-broken implants (No. 3–5, 7; 45 % of the measured values are ≤1 mm and 75 % are ≤1.5 mm). The broken implants (No. 1, 2, and 6) showed step formation >2 mm that is most probably caused by a lateral lifting of the implant during the tightening of the SmartNails®. None of the implants met that the CPC part should not protrude into the cartilage layer (Table 5). The nature of the implants in terms of size and shape, specifically the proportion and the shape of the CPC part, plays a critical role for the success of implantation. Implants with a thin/flat CPC part have a higher risk to break during SmartNail® tightening if they do not perfectly fit into the defect; this effect is amplified when the CPC part is additionally weakened by the presence of two channels (implant No. 1, broken; implant No. 3, broken in the first of two trials). In addition, in case of implant No. 2, the lower resolution of printing with the 410 μm nozzle and the placement of the channel caused an adverse reduction of the CPC part (‘loss of information’ form the virtual to the real object) at the bottom of the defect which in turn may have contributed to imperfect fitting and negative step values (Table 5). In addition, the reconstruction of the cartilage was good in terms of volume and integration of the hydrogel with the adjacent cartilage. 70 percent (5/7) of the implants achieved more than 85 % of the possible MOCART score points. In two implants, the cartilage layer was insufficiently (No. 2 and 6). These were also the two implants that had the greatest overall inaccuracy of fit and were broken. As a result, integration with the surrounding cartilage tissue was reduced. Challenges also exist with the necessary lateral protrusion of the hydrogel layer over the CPC portion (sole hydrogel portion without CPC with conical defect geometry in the model).

The SmartNail® fixation worked very well in all cases – the implants were held in the defect and even just one SmartNail® was sufficient for stable anchoring. This statement is limited by the fact that the anchorage was not tested under real conditions, i.e. when the knee was moved. However, the insertion of the SmartNails® carries the risk of CPC fracture, e.g. due to tilting during insertion or if an improperly fitting implant is tightened. A replacement implant should therefore be available. A limitation of the SmartNail® fixation is that it is not possible to drill the channels within the CPC implant, which requires a prior definition and does not allow an intra-operative decision on the exact SmartNail® placement (and number) depending on the bone quality.

There are several limitations in our proof-of-concept study that may have affected the outcome. First, artificially in this setup with body donors was the defect creation with a chisel in a ‘healthy’ osteochondral tissue that resulted in a very rough bone surface within the defect; small protrusions may not have been detected with the low-resolution clinical MRI. In addition, visualization of the implants in MRI was challenging in the donor knees due to air in the joint space, which would not occur *in vivo;* better contrast between the implant surface, i.e. the hydrogel, and the joint space is expected *in vivo*. Finally, the number of measurement points to assess the fit was limited and their position arbitrarily determined. In an ongoing study, we aim to digitalize the whole process to develop a quality control of the complete process chain. Therefore, we are developing a methodology to evaluate each step of the entire process chain by superimposition of pre- and postoperative models, including models of the manufactured implants obtained from pre-implantation scans.

## 5 Conclusion

In this work, the general applicability of the MRI-guided workflow for the additive manufacturing of patient-specific implants with zonal design for the regeneration of osteochondral defects under near-clinical conditions was demonstrated and critical points of the approach were defined. The inherent limitations in the resolution of both the MRI with clinically used settings and the extrusion-based 3D printing technology led to inaccuracies in implant fitting (‘missing information’ from the defect and ‘loss of information’ from the virtual model to the real object). Therefore, tolerances in the implant design as well as flexibility for intraoperative adjustments are necessary and – as shown in the work – possible. Anchorage of the implants in the defect was successfully achieved with SmartNail® fixation. The clinical application of this approach can only be recommended for larger defects (>2 cm^2^), as small defect geometries – flat defects with a small amount of bone – are more difficult to print and anchor while the manufactured implants have a higher tendency to inaccuracy and implant failure due to fracture.

## CRediT authorship contribution statement

**Max von Witzleben:** Writing – review & editing, Writing – original draft, Visualization, Methodology, Investigation, Formal analysis, Data curation, Conceptualization. **Suihong Liu:** Writing – review & editing, Visualization, Methodology, Investigation, Formal analysis, Data curation, Conceptualization. **Philipp Sembdner:** Writing – review & editing, Writing – original draft, Visualization, Methodology, Investigation, Formal analysis, Data curation, Conceptualization. **Stefan Holtzhausen:** Writing – review & editing, Resources, Methodology, Investigation, Conceptualization. **Sophia Freya Ulrike Blum:** Writing – review & editing, Writing – original draft, Visualization, Resources, Methodology, Investigation, Formal analysis, Data curation, Conceptualization. **Jörg Lützner:** Writing – review & editing, Methodology, Conceptualization. **David Kilian:** Writing – review & editing, Methodology, Conceptualization. **Ute Nimtschke:** Writing – review & editing, Resources, Methodology. **Michael Gelinsky:** Writing – review & editing, Resources, Funding acquisition. **Anja Lode:** Writing – review & editing, Writing – original draft, Visualization, Methodology, Investigation, Conceptualization. **Henriette Bretschneider:** Writing – review & editing, Writing – original draft, Visualization, Resources, Methodology, Investigation, Funding acquisition, Data curation, Conceptualization.

## Ethics statement

The study using human body donors was approved by the ethics committee of Technische Universität Dresden (BO-EK-50012023).

## Declaration of competing interest

The authors declare that they have no known competing financial interests or personal relationships that could have appeared to influence the work reported in this paper.

## Data Availability

Data will be made available on request.
