# Supplementary material for: Additive manufacturing of patient-specific, biphasic implants with zonal design for regeneration of osteochondral defects–critical evaluation of the work flow from clinical MRI data to implantation
Source: Mater Today Bio. 2025 May 13;32:101858. doi: 10.1016/j.mtbio.2025.101858 (PMC12145557; doi:10.1016/j.mtbio.2025.101858)
Supplement: Multimedia component 1 [file mmc1.docx]

Supplementary

**Additive manufacturing of patient-specific, biphasic implants with zonal design for regeneration of osteochondral defects – critical evaluation of the work flow from clinical MRI data to implantation**

Max von Witzleben^1#^, Suihong Liu^1#§^, Philipp Sembdner^2#^, Stefan Holtzhausen^2#^, Sophia Freya Ulrike Blum^3#^, Jörg Lützner^4^, David Kilian^5§^, Ute Nimtschke^6^, Michael Gelinsky^1^, Anja Lode^1*^, Henriette Bretschneider^1,4#^*

^1^Centre for Translational Bone, Joint and Soft Tissue Research, Faculty of Medicine and University Hospital *Carl Gustav Carus*, Technische Universität Dresden, Dresden, Germany

^2^Institute of Machine Elements and Machine Design, Faculty of Mechanical Engineering, Technische Universität Dresden, Dresden, Germany

^3^Institute and Polyclinic for Diagnostic and Interventional Radiology, University Hospital *Carl Gustav Carus*, Technische Universität Dresden, Dresden, Germany

^4^University Center of Orthopaedics, Trauma and Plastic Surgery, University Hospital *Carl Gustav Carus*, Technische Universität Dresden, Dresden, Germany

^5^Department of Materials Science and Engineering, Stanford University, Stanford, CA, USA

^6^Institute of Anatomy, Faculty of Medicine and University Hospital *Carl Gustav Carus*, Technische Universität Dresden, Dresden, Germany

^#^these authors contributed equally to this work

*corresponding authors: [henriette.bretschneider@uniklinikum-dresden.de](mailto:henriette.bretschneider@uniklinikum-dresden.de), [anja.lode@tu-dresden.de](mailto:anja.lode@tu-dresden.de)

^§^current address:

David Kilian

Department of Complex Tissue Regeneration (CTR), MERLN Institute for Technology-Inspired Regenerative Medicine, Maastricht University, Maastricht, The Netherlands

Suihong Liu

Engineering Science and Mechanics Department and The Huck Institutes of Life Science, Penn State University, University Park, PA, USA

**Prefrabricated support structures**

Stereolithographic (SLA) printing is renowned for its high resolution and fine detail, achieved through a precisely focused laser that cures photopolymer resins layer-by-layer. The effective resolution in the XY plane is governed by the beam’s focal diameter and the optical system, typically yielding feature sizes in the hundreds of microns, while the z-resolution is determined by the chosen layer thickness—often as low as 25 µm. However, the final dimensional accuracy of a print is significantly influenced by post-processing steps. During washing in solvents such as isopropyl alcohol and subsequent UV post-curing, additional polymerization and thermal contraction can induce small distortions and localized warping. These effects are particularly evident near support structures and in areas with large, flat surfaces, where differential shrinkage and peeling forces may slightly alter the intended geometry. Consequently, careful optimization of post-processing conditions and the minimal usage of support structures were esential to preserve the high resolution and minimize minor distortions (< 500 µm).

Each defect was printed in two parts: a base plate with a defined position of one (or more) implant design(s) and a second part unique to each defect. The second part tighly fit onto the specific implant design and was secured with tape (**Figure S1**). Only liquids like water and crosslinking agents, were able to leak into the narrow gap between both parts. The base plate was placed on the printing bed of the GeSiM BioScaffolder and secured with additional tape. After the origin of the baseplate was configured within the GeSiM Software each implant design became printable. However, small distortions and minor misplacements of less than 500 µm were inevitable.


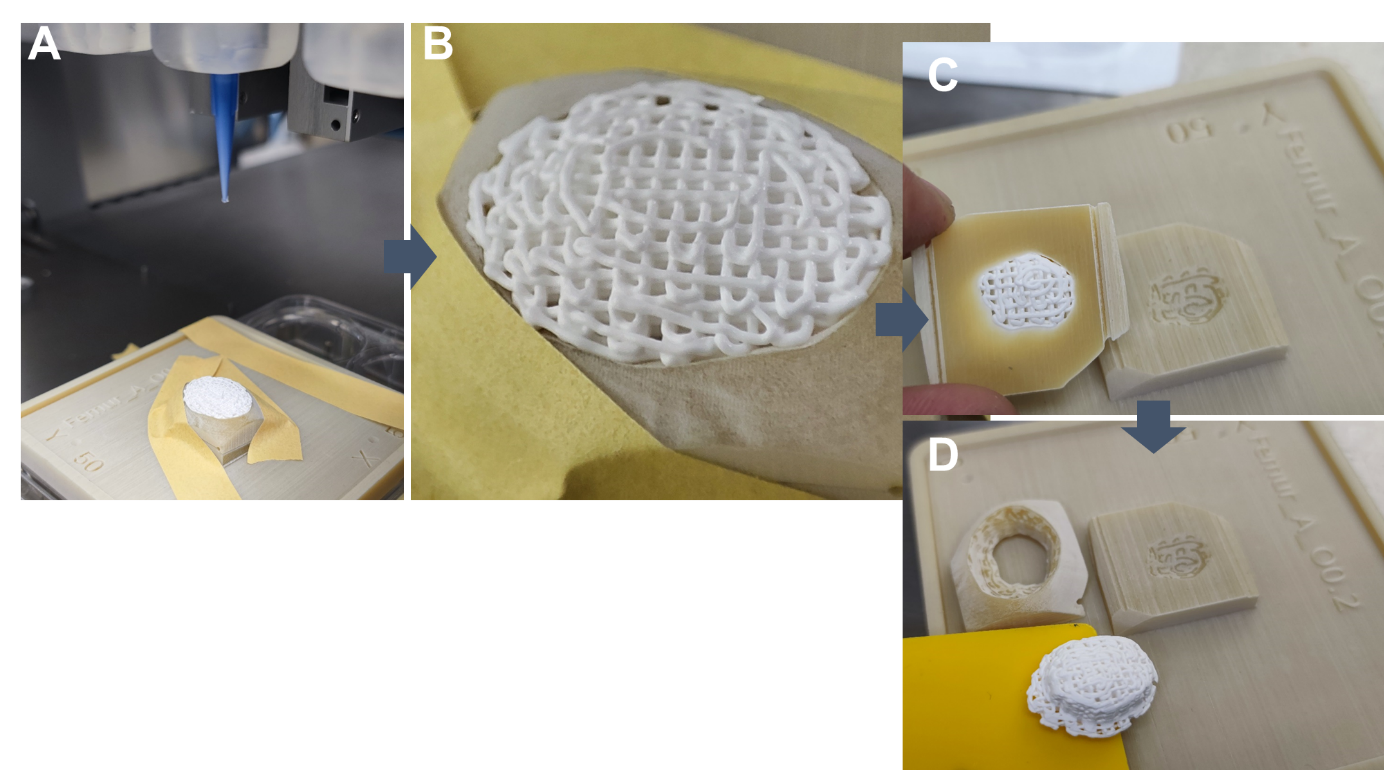


Figure S1: Display of the prefabricated support structures and their use in the printing process. After configuring them within the printer environment and printing into them (A, B), the fixation tape can be removed to ensure a full setting of the CPC (C), so that the implant can be removed from the support structure (D).
